# Roles and Molecular Mechanisms of Physical Exercise in Sepsis Treatment

**DOI:** 10.3389/fphys.2022.879430

**Published:** 2022-06-29

**Authors:** You Wu, Xiaofeng Guo, Yuliang Peng, Zongping Fang, Xijing Zhang

**Affiliations:** ^1^ Department of Intensive Care Unit, Xijing Hospital, The Fourth Military Medical University, Xi’an, China; ^2^ Department of Intensive Care Unit, Joint Logistics Force No. 988 Hospital, Zhengzhou, China

**Keywords:** physical exercice, sepsis, organ failure, outcome, molecular mechanism

## Abstract

Physical exercise is a planned, purposeful action to keep a healthy lifestyle and improve physical fitness. Physical exercise has been widely used as a non-pharmacological approach to preventing and improving a wide range of diseases, including cardiovascular disease, cancer, metabolic disease, and neurodegenerative disease. However, the effects of physical exercise on sepsis have not been summarized until now. In this review, we discuss the effects of physical exercise on multiple organ functions and the short- and long-time outcomes of sepsis. Furthermore, the molecular mechanisms underlying the protective effects of physical exercise on sepsis are discussed. In conclusion, we consider that physical exercise may be a beneficial and non-pharmacological alternative for the treatment of sepsis.

## 1 Introduction

Sepsis is defined as a life-threatening organ failure caused by a dysregulated host response to infection and affects approximately 19.4 million individuals each year (Prescott and Angus, 2018). In recent years, there have been several interventions utilized to improve the survival of patients with sepsis. As a result, the mortality of in-hospital sepsis patients has declined, from 35% to 18%, making for many sepsis survivors (Kaukonen et al., 2014; Prescott and Angus, 2018). However, emerging data suggest that one-third of the survivors die within a year, and one-sixth have clinical sequelae including cognitive dysfunction, physical incapacity, exacerbation of chronic medical conditions, and mental problems (Iwashyna et al., 2010; Yende et al., 2014; Prescott and Angus, 2018; Venet and Monneret, 2018). The reasons for poor long-term outcomes after sepsis are complex and include residual organ damage. During sepsis, multiple organ systems, including the respiratory, renal, cardiovascular, neurological, hepatic, and hematological systems, are typically impaired simultaneously, resulting in poor clinical outcomes (Lelubre and Vincent, 2018). Multiple organ failure may remain despite successful treatment for sepsis. Therefore, effective interventions that target multiple organ systems are critical for improving the short- and long-time outcomes of sepsis.

Physical exercise is a planned, purposeful action to maintain a healthy lifestyle and improve physical fitness (WHO, 2010). Physical exercise has been widely used as a non-pharmacological approach to preventing and improving a wide range of diseases, including cardiovascular disease, cancer, metabolic disease, and neurodegenerative disease (Gleeson et al., 2011; Kim et al., 2014). For example, the obesity-associated metabolic disease was improved by moderate- or high-intensity exercise (Wang et al., 2017). In addition, physical exercise was able to inhibit cancer metastasis, ameliorate the side effects of cancer treatment, and prevent cancer-related death. Furthermore, there is emerging evidence that physical exercise acts on multiple organ systems under various conditions (Sabaratnam et al., 2022). However, the effects of physical exercise on sepsis have not been summarized until now. This review outlines the effects of physical exercise on multiple organ functions and the short- and long-term outcomes of sepsis. To clarify the role of physical exercise in sepsis, it is crucial to understand the molecular mechanisms mediating the protective impacts of physical exercise. Therefore, the molecular mechanisms underlying the protective effects of physical exercise on sepsis are also discussed.

## 2 Effects of Physical Exercise on Multiple Organ Function and the Outcomes of Sepsis

### 2.1 Effects of Physical Exercise on Cardiovascular Function

The cardiovascular system is frequently impaired in sepsis. Cardiovascular dysfunction is characterized by a total decrease in left ventricular diastolic and systolic functions, which leads to arterial hypotension (Rong et al., 2021). Sepsis patients with cardiovascular dysfunction have a higher mortality rate than those with normal cardiovascular function during hospitalizations (Merx and Weber, 2007). After hospitalizations, sepsis survivors have a 13-fold increased risk of cardiovascular events compared with survivors of other diseases (Yende et al., 2014). Therefore, cardiovascular dysfunction is the leading problem in sepsis patients during and after hospitalizations.

Several studies have demonstrated that physical exercise promotes metabolic flexibility, myocardial remodeling, and angiogenesis, which have been considered to prevent and treat cardiovascular dysfunction in various diseases (Wu et al., 2019a). Mehanna et al. (2007) demonstrated that exercise preconditioning attenuated the alterations in arterial pressure and heart rate of Wistar rats at 5 h following lipopolysaccharide (LPS) injection, suggesting that exercise training alleviated cardiovascular abnormalities during sepsis. Similarly, Chen et al. (2007) showed that exercise-trained rats had lower basal levels of heart rate and arterial pressure, as well as less severe cardiac injury at 72 h following LPS treatment. This study also found that exercise training before sepsis reduced plasma levels of pro-inflammatory cytokines and nitrate, which are potential mechanisms of the positive effects of physical exercise on cardiovascular function in sepsis (Chen et al., 2007). Furthermore, cardiovascular function measured by ejection fraction after sepsis was alleviated by exercise preconditioning (Sun et al., 2020; Khoshkhouy et al., 2021). Overall, these animal studies suggest that cardiovascular dysfunction may be ameliorated by physical exercise preconditioning in sepsis.

### 2.2 Effects of Physical Exercise on Renal Function

Septic patients often develop uropenia with increased serum creatinine and urea. Those who meet consensus criteria for acute kidney injury (AKI) are deemed to have sepsis-associated AKI. A survey suggested that over 60% of patients with sepsis have AKI (Poston and Koyner, 2019). Sepsis patients with AKI have a higher mortality rate than patients without AKI. Therefore, AKI has been long-regarded as an independent risk factor of mortality in sepsis during hospitalization (Poston and Koyner, 2019). Furthermore, a study involving 2,617 sepsis survivors revealed that they have a 2.7-fold increased risk of readmission for AKI compared with survivors for other diseases (Prescott and Angus, 2018). Here, we investigate whether physical exercise acts on AKI in sepsis.

In an ischemic-reperfusion model, physical exercise can prevent and attenuate renal dysfunction in healthy individuals (de Lima et al., 2019). In gentamicin-associated acute kidney injury, physical exercise promotes the recovery of renal structure and function by restoring redox balance (Oliveira et al., 2017). Interestingly, several studies have shown that exhaustive exercise is associated with kidney injury (Wu et al., 2012; Hosoyamada et al., 2016; Gundlapalli et al., 2021). In mice with sepsis, the impairment of kidney tubules is less severe with physical exercise (Sossdorf et al., 2013). In contrast, Húngaro et al. (2020) found that physical exercise increased the renal tubulointerstitial space and expression levels of NGAL, a gene related to kidney injury, and TLR4, suggesting that physical exercise enhances renal dysfunction after LPS treatment. Therefore, the effects of physical exercise on renal function are unclear and may depend on the intensity and duration of physical exercise.

### 2.3 Effects of Physical Exercise on Neurological Function

Sepsis-associated encephalopathy is one of the most common complications in sepsis. Approximately 70% of septic patients suffer consciousness, delirium, concentration deficiency, anxiety, depression, and cognitive dysfunction during hospitalization (Molnár et al., 2018). About 50% of sepsis survivors acquire long-time cognitive dysfunction, including deficiency in memory, attention, executive function, verbal skills, and mental problems after hospitalization (Davydow et al., 2012; Molnár et al., 2018). Moreover, sepsis-associated encephalopathy is responsible for poor sepsis outcomes resulting in high hospitalization costs. Therefore, it is essential to prevent and treat neurological dysfunction during sepsis.

There is ample evidence that physical exercise alleviates structural brain abnormalities and cognitive dysfunction in a wide range of brain diseases, including Alzheimer’s disease, Huntington’s disease, and Parkinson’s disease (Gubert and Hannan, 2021). Physical exercise enhances neuroplasticity, neurogenesis, angiogenesis, and synaptic activity to improve brain structure and function in various brain disorders (Sujkowski et al., 2022). In relation to traumatic brain injury, Morris et al. (2016) reported that physical exercise improved cognitive dysfunction. In sepsis, the endocannabinoid system and cyclooxygenase enzyme play central roles in cognitive dysfunction by regulating neuroinflammation. Moosavi Sohroforouzani et al. (2020) found that the escape distance and latency to reach the platform in the LPS treatment group were longer than those in the LPS+ treadmill aerobic exercise group, and exercise preconditioning reduced cannabinoid receptor 2 receptor levels as well as cyclooxygenase-2 levels, suggesting that treadmill aerobic exercise had a beneficial effect on cognitive function by regulating the endocannabinoid system and cyclooxygenase in sepsis. In *Trypanosome cruzi* infection, exercise preconditioning decreases the parasite peak and contributes to the survival of neurons and neuronal hypertrophy (Moreira et al., 2014). These results show that exercise preconditioning ameliorates neurological dysfunction in sepsis.

### 2.4 Effects of Physical Exercise on Other Organ Functions

As discussed above, physical exercise preconditioning has protective effects on cardiovascular and neurological functions in sepsis. Here, we discuss whether physical exercise improves other organ functions in sepsis. de Araújo et al. (2012) firstly found that physical activity reduced the static elastance of the lung, alveolar collapse, lung collagen and fiber content, and neutrophil levels in bronchoalveolar lavage fluid. Subsequent studies verified that pulmonary surfactant function was impaired; neutrophil influx in the liver and lung, capillary plugging, and expression levels of lung interleukin 6 (IL-6) were increased in sepsis, but voluntary running reversed these septic responses (Tyml et al., 2017). Similarly, preconditioning exercise prevented aggravations of lung injury by mediating purinergic system and oxidative stress under septic condition (Miron et al., 2019). These animal studies suggest that lung and liver functions can be improved by exercise preconditioning during sepsis. In addition, Al-Nassan and Fujino (2018) demonstrated that a mild exercise preconditioning could preserve muscle mass and prevent atrophy during sepsis. Furthermore, exercise preconditioning increased survival, ameliorated multiple organ damage, and recovered pro- and anti-inflammatory balance by modifying gut microbiota composition (Kim and Kang, 2019).

Overall, the above findings indicate that exercise preconditioning protects against multiple organ failure during sepsis in experimental models. Clinical research demonstrates that early physical rehabilitation in septic patients might improve physical function and reduce the inflammatory response at 6–12 months post-hospital discharge (Kayambu et al., 2011; Kayambu et al., 2015; Ahn et al., 2018). Therefore, physical exercise may be a non-pharmacological method to improve multiple organ dysfunction in sepsis.

### 2.5 Effects of Physical Exercise on the Outcomes of Sepsis

Here, we discuss whether physical exercise affects the outcomes of sepsis. Based on experimental models, several studies have suggested that regular exercise alters the morbidity of sepsis and increases the survival rate (Sossdorf et al., 2013; Kim and Kang, 2019; Wang et al., 2021). In a clinical study, Wang et al. (2014) indicated an association between physical exercise preconditioning and susceptibility to sepsis. They concluded that individuals with low rates of physical exercise and high rates of watching television presented with higher morbidity and mortality of sepsis (Wang et al., 2014). However, sepsis survivors have a significant reduction in exercise capacity and physical activity that may continue even 3 months after hospitalization (Borges et al., 2015), and little information is available regarding the effects of post-hospital exercise on the long-term outcomes of sepsis.

Although the effects of physical exercise in improving organ function of sepsis are different in different organ systems, several studies show that exercise preconditioning can ameliorate sepsis-mediated multiple organ failure and reduce morbidity and mortality of sepsis (summarized in Table 1). In conclusion, we consider that physical exercise preconditioning may be a beneficial and non-pharmacological alternative for preventing and treating sepsis and is suitable for any individual.

**TABLE 1:** Effects of physical exercise on multiple organ function and outcomes of sepsis.

Organ	Effects	Molecular mechanisms	Reference
Heart	Attenuate the alterations in arterial pressure and heart rate	—	Mehanna et al. (2007)
	Attenuate basal levels of heart rate, arterial pressure and cardiac injury	Reduce levels of pro-inflammatory cytokines and nitrate	Chen et al. (2007)
	Ameliorate cardiac injury	Reduce levels of pro-inflammation, oxidative stress and apoptosis	Khoshkhouy et al. (2021)
	Ameliorate cardiovascular dysfunction reflected by ejection fraction	Inhibit GCN2-eIF2α/ATF4 pathway	Sun et al. (2020)
Kidney	Ameliorate kidney tubular damage	Increase lysophosphatidylcholines and decrease inflammatory cytokines	Sossdorf et al. (2013)
	Expand the renal tubulointerstitial space	Increase levels of NGAL and TLR 4	Húngaro et al. (2020)
Brain	Reduce escape distance and latency to arrive the platform	Inhibit endocannabinoid system and COX	Moosavi Sohroforouzani et al. (2020)
	Contribute to survival of neuron and neuronal hypertrophy	Increased levels of TGF-β and TNF-α	Moreira et al. (2014)
Lung	Enhance pulmonary surfactant function	Reduce levels of pro-inflammation and neutrophil influx in lung	Tyml et al. (2017)
	Ameliorate lung injury	Reduce density of purinergic enzymes and receptors, and oxidative stress	Miron et al. (2019)
	Ameliorate pulmonary edema	Decrease levels of pro-inflammation and restore redox balance	Wang et al. (2021)
	Reduce static elastance of lung and alveolar collapse	Decrease content of lung collagen and fiber, levels of neutrophils in BALF	de Araújo et al. (2012)
Liver	—	Reduce neutrophil influx in liver	Tyml et al. (2017)
	Make no effect on liver damage	—	Sossdorf et al. (2013)
Skeletal muscle	—	Reduce capillary plugging and increase eNOS	Tyml et al. (2017)
	Preserve muscle mass and prevent atrophy	—	Al-Nassan and Fujino (2018)
Outcomes	Alter the morbidity of sepsis and increase the survival rate of sepsis	Modify gut microbiota	Sossdorf et al. (2013), Kim and Kang (2019), Kayambu et al. (2011), Wang et al. (2014), Ahn et al. (2018), Wang et al. (2021)

## 3 Molecular Mechanisms of Physical Exercise in Sepsis Treatment

### 3.1 Mitochondrial Quality Control

#### 3.1.1 Mitochondrial Biogenesis

Mitochondrial quality is controlled by various processes such as mitochondrial biogenesis, mitochondrial fusion/fission, and mitophagy. Mitochondrial biogenesis contributes to the production of new mitochondria and mitochondrial content. These processes are controlled by biogenesis signals, such as PGC-1α, NRF-1, NRF-2, AMPK, SIRT1, and TFAM. PGC-1α plays a central role in mitochondrial biogenesis and is activated by the SIRT1-AMPK pathway, which then interacts with NRF-1 and NRF-2 in both the mitochondria and nucleus (Song et al., 2021). In the mitochondria, PGC-1α binds to NRF-1 and NRF-2, coactivating TFAM, which in turn mediates mitochondrial DNA translation, transcription, and replication (Song et al., 2021). In the nucleus, PGC-1α binding to NRF-1 and NRF-2 induces nuclear translocation of mitochondrial proteins, which are then imported into the mitochondria (Song et al., 2021). During sepsis, the expression levels of PGC-1α, TFAM, NRF-1, and NRF-2 are increased in multiple organ tissues, including the liver, heart, brain, and lungs, in the initial stage and decreased in the late stage (Rayamajhi et al., 2013; Vanasco et al., 2014; Wu et al., 2019b). Haden et al. (2007) first demonstrated that mitochondrial biogenesis induction could restore basal metabolism in *Staphylococcus* aureus sepsis. Thereafter, MacGarvey et al. (2012) showed that targeted induction of mitochondrial biogenesis could attenuate multiple organ dysfunction in sepsis. In addition, several studies have repeatedly verified that PGC-1α overexpression attenuates multiple organ dysfunction in sepsis (Tran et al., 2011; Yi et al., 2020; Li et al., 2021). Various proteins of mitochondrial biogenesis have been found to be increased after exercise. A systematic review showed that physical exercise increased the expression levels of PGC-1α, NRF-1, NRF-2, and TFAM and promoted mitochondrial biogenesis in Parkinson’s disease (Nhu et al., 2021). In addition, Zhang and Gao (2021) found that physical exercise protects against cardiovascular disease by promoting mitochondrial biogenesis. Therefore, physical exercise could enhance multiple organ functions through the induction of mitochondrial biogenesis.

#### 3.1.2 Mitochondrial Dynamics

Mitochondrial fusion and fission regulate mitochondrial number and size. These processes are mediated by the fission proteins, Drp1 and Fis1, and the fusion proteins, Mfn2, Mfn1, and OPA1. In mitochondrial fusion, homo- and hetero-oligomeric structures are formed by Mfn1 and Mfn2 to link two neighboring mitochondria for outer membrane fusion, and OPA1 directly promotes inner membrane fusion (Chan, 2012). During mitochondrial fission, Drp1 translocates from the cytosol to the mitochondria and forms Drp1 complexes to constrict the mitochondrial tubule. The parent mitochondria are then segregated into two daughter mitochondria (Losón et al., 2013). In sepsis, the fusion proteins Mfn2 and OPA1 are decreased, and the fission protein Drp1 is increased in the liver, heart, and immune cells (Gonzalez et al., 2014; Shen et al., 2018). Inhibition of Drp1 and overexpression of Mfn2 improve organ dysfunction and poor outcomes in sepsis (Gonzalez et al., 2014; Deng et al., 2018; Wu et al., 2019b). Jang et al. (2018) found that physical exercise enhanced the expression of Mfn2, OPA1, and p-Drp1 Ser637 and balanced mitochondrial fusion and fission. In addition, treadmill exercise enhances learning skills and memory in Alzheimer’s disease by balancing mitochondrial fusion and fission (Yan et al., 2019).

#### 3.1.3 Mitophagy

**FIGURE 1:**
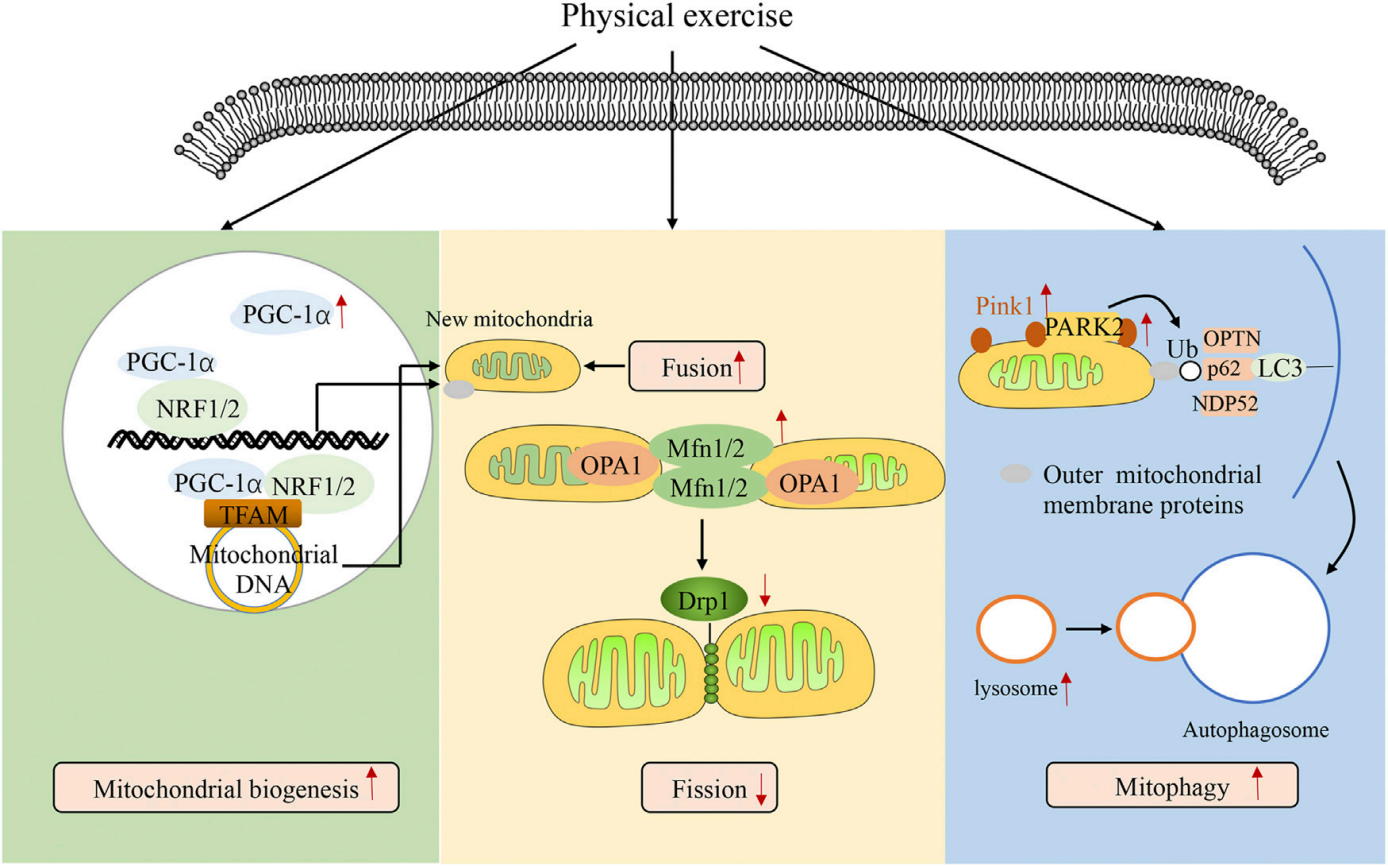
Physical exercise regulating mitochondrial quality control. The figure shows how physical exercise mediates mitochondrial quality control. Mitochondrial quality is controlled by various processes, including mitochondrial biogenesis, mitochondrial fusion/fission, and mitophagy. Processes of mitochondrial biogenesis are controlled by biogenesis signals such as PGC-1α, NRF-1, NRF-2, AMPK, SIRT1, and TFAM. PGC-1α plays a central role in mitochondrial biogenesis, interacting with NRF-1 and NRF-2 in both the mitochondria and nucleus. In the mitochondria, PGC-1α binds with NRF-1 and NRF-2, coactivating TFAM, which in turn mediates mitochondrial DNA translation, transcription, and replication. In the nucleus, PGC-1α binds with NRF-1 and NRF-2, inducing the nuclear translation of mitochondrial proteins, which are imported into the mitochondria. Mitochondrial fusion and fission are mediated by fission proteins such as Drp1 and fusion proteins such as Mfn2, Mfn1, and OPA1. PINK1 import to the inner mitochondrial membrane is inhibited when it detects a damaged mitochondrion, resulting in the accumulation of PINK1 on the outer mitochondrial membrane. PINK1 phosphorylates ubiquitin, a substrate of PINK1, which then induces the recruitment of Parkin to the damaged mitochondria. Then, PARK2 is phosphorylated and binds to outer mitochondrial membrane proteins and autophagy adaptor proteins, ultimately resulting in mitophagy. Physical exercise promotes mitochondrial quality control.

Mitophagy is the selective elimination of aged and damaged mitochondria, which can help maintain mitochondrial homeostasis. The import of PINK1 to the inner mitochondrial membrane is blocked when a damaged mitochondrion is detected, resulting in the accumulation of PINK1 on the outer mitochondrial membrane. PINK1, which is activated through auto-phosphorylation, can phosphorylate ubiquitin, a substrate of PINK1, which then induces the recruitment of Parkin to damaged mitochondria. After that, PARK2 is activated by phosphorylation, which binds to the outer mitochondrial membrane and autophagy adaptor proteins, including OPTN and NDP52, ultimately resulting in autophagosomes (Lazarou et al., 2015). Finally, autophagosomes fuse with a lysosome, degrading damaged mitochondria. In sepsis, mitophagy is induced in the initial stage, but lysosomal degradation is impaired in the late stage, leading to multiple organ dysfunction (Chien et al., 2011; Hsieh et al., 2011). Knockdown of PINK1 or PARK2 exacerbates multiple organ dysfunction during sepsis (Kang et al., 2016). These suggest that complete induction of mitophagy presents as a therapeutic target during sepsis. There is evidence that physical exercise enhances the recruitment of PARK2 to the outer mitochondrial membrane to stimulate mitophagy in cardiovascular disease (Wu et al., 2019a; Memme et al., 2021). Furthermore, Hwang et al. (2018) demonstrated that physical exercise reduced the expression levels of P62 and enhanced the expression of LAMP2 and cathepsin L, suggesting that physical exercise promotes lysosomal degradation. Therefore, physical exercise could reverse sepsis-induced disruption of the lysosomal degradation and promote complete induction of mitophagy. Collectively, previous results have suggested that physical exercise improves organ dysfunction by regulating mitochondrial quality control (Figure 1).

### 3.2 Systemic Inflammation

Sepsis is characterized by hyperinflammatory responses and immunosuppression in the initial and late stages of the disease, respectively. Hyperinflammatory responses are the leading cause of organ dysfunction. During sepsis, innate immune cells recognize pathogen-associated molecular patterns *via* pattern recognition receptors, activating numerous signaling pathways in the cell (Cecconi et al., 2018). Activation of these pathways results in the downstream activation of MAP3K7, which then activates the JNK-p38-ERK pathways, IRFs, and NF-κB (Lawrence, 2009). Finally, inflammatory cytokines, including IL-6, IL-12, TNF-α, and IL-1β, are released, inducing endothelial dysfunction and cell damage in multiple organ tissues. Damage-associated molecular patterns produced by tissue injury have the same function as pathogen-associated molecular patterns and amplify immune responses (Timmermans et al., 2016). These factors induce multiple organ dysfunction in sepsis.

Numerous studies have shown that physical exercise improves organ dysfunction by reducing systemic inflammation in sepsis patients. Wang et al. (2021) found that aerobic exercise decreased lung neutrophil content and the mRNA expression levels of IL-6, TNF-α, Glu1, CXCL-1, and HMGB1 in the lung to improve respiratory dysfunction. Shimojo et al. (2019) showed that swimming decreased serum inflammatory cytokines and increased anti-inflammatory cytokines by decreasing dopamine. Miron et al. (2019) demonstrated that physical exercise decreases serum IL-6 and IL-1β expression following LPS treatment. Tyml et al. (2017) showed that voluntary running protects against respiratory dysfunction, hepatic dysfunction, and neutrophil influx by reducing inflammation in sepsis. Collectively, these studies conclude that physical exercise improves organ dysfunction by reducing systemic inflammation in sepsis.

### 3.3 Redox Balance

Oxidants and antioxidants are involved in various diseases. The oxidative burst promotes the production of reactive oxygen species (ROS) and reactive nitrogen species. To maintain cellular homeostasis, antioxidant enzymes, including glutathione peroxidase, superoxide dismutase, and catalase, act as oxidant scavengers and decrease the cellular level of oxidants (Mantzarlis et al., 2017). In the past decades, several studies have suggested that ROS are induced during sepsis and involved in the development of sepsis-induced multiple organ dysfunction (Jung et al., 2000; Pleiner et al., 2003; Ritter et al., 2003). A clinical study showed that the antioxidant potential was increased to a greater extent in sepsis survivors than in non-survivors (Cowley et al., 1996). Further research verified that the balance between oxidants and antioxidants was disrupted in sepsis, resulting in oxidative stress, cell death, and organ injury (Miliaraki et al., 2022).

Converging studies have suggested that ROS are involved in mediating the effects of physical exercise. Adams et al. suggested that physical exercise decreased ROS generation, resulting in improving acetylcholine-mediated vasodilatation and reducing Ang II-mediated vasoconstriction (Adams et al., 2005). In addition, Miron et al. (2019) found that physical exercise reduces lung lipid peroxidation and reactive species. Furthermore, Wu et al. (2020) demonstrated that physical exercise alleviated the increased ROS levels and apoptosis in kidney tissues. However, Mendonça et al. (2019) found that pre-infection exercise aggravates acute infections by aggravating oxidative stress. A review summarized that prolonged endurance exercise promoted oxidative stress, whereas moderate physical exercise reduced oxidative stress (Gomez-Cabrera et al., 2021). Therefore, physical exercise is considered a double-edged sword for redox balance, depending on the intensity and duration of physical exercise.

### 3.4 Gut Microbiome

There are trillions of microbiota in the human gastrointestinal tract that play diverse roles in health and disease. Recent breakthroughs in technology, such as metagenome and 16S ribosomal RNA sequencing, have enabled progress in understanding the gut microbiome. This has led to an enormous increase in research elucidating the association between the gut microbiome and diseases. In sepsis, a study revealed that the levels of beneficial *Lactobacillus* and *Bifidobacterium* were decreased, and the abundance of pathogenic *Pseudomonas* and *Staphylococcus* was increased (Shimizu et al., 2006). Disruption of the gut microbiome at both the functional and compositional levels promoted multiple organ dysfunction in patients with sepsis (Liu et al., 2019). Moreover, disruption of the gut microbiome increased the susceptibility of rats to sepsis (Haak and Wiersinga, 2017). It also reported that intervention with three microbiota-derived short-chain fatty acids could improve multiple organ dysfunction in sepsis (Haak and Wiersinga, 2017). These new insights suggest that the gut microbiome plays an essential role in mediating sepsis-induced multiple organ dysfunction.

There is evidence that exercise may affect the gut microbiome, which can then modulate multiple organ dysfunction in sepsis. For example, physical exercise changes the composition of the gut microbiome, including an increase in the abundance of beneficial *Lactobacillus* and *Bifidobacterium* (Queipo-Ortuño et al., 2013). Modifying the composition of the gut microbiome by exercise preconditioning can increase survival, ameliorate multiple organ damage, and restore pro- and anti-inflammatory balance in sepsis (Kim and Kang, 2019). Physical exercise also increases short-chain fatty acid levels in both humans and rodents, which is beneficial for multiple organ dysfunction in sepsis (Allen et al., 2018). Physical exercise enhances SCFA levels by increasing SCFA-producing bacteria, including the propionate producer *Propionibacterium freudenreichii* and the butyrate producers *Faecalibacterium prausnitzii* (Húngaro et al., 2020; Ramos et al., 2022)*.* Furthermore, physical exercise increases the diversity of the gut microbiome and decreases gut transit time. Therefore, the gut microbiome may be a bridge between physical exercise and sepsis.

### 3.5 Noncoding RNAs

Non-coding RNA (ncRNA) is a class of RNA molecules that cannot encode proteins or peptides, mainly including microRNA (miRNA), long non-coding RNA (lncRNA), circular RNA (circRNA), and small interfering RNA (siRNA) (Matsui and Corey, 2017). ncRNA binds to many molecular targets to form a regulatory network, initiating specific cellular biological responses. In addition, ncRNA can regulate gene expression, influence intracellular signaling, and participate in epigenetic modifications, thus playing a crucial role in various disease (Matsui and Corey, 2017). Many studies have demonstrated that multiple miRNAs, such as mi-R210, miR-23b, and miR-29a, can suppress NF-κB and IL-6 expression in sepsis by regulating the function of the immune cells (Qi et al., 2012; Benz et al., 2016). In addition, a study showed that lncRNA HOTAIR regulates cardiomyocyte TNF-α synthesis in a murine sepsis model (Wu et al., 2016). Furthermore, recent research suggested that mcircRasGEF1B protected cells from infection by regulating the stability of mature ICAM-1 mRNAs (Ng et al., 2016). In conclusion, there is growing evidence that ncRNA is involved in regulating pathophysiological processes in sepsis.

Physical exercise has been reported to regulate various ncRNA, including circulating miRNAs (Baggish et al., 2011). For example, exercise training increased cell proliferation *via* downregulating the levels of miR-135a (Improta-Caria et al., 2020). In the traumatic brain injury model, physical exercise could attenuate cognitive dysfunction *via* upregulating the levels of miR-21 (Hu et al., 2015). Interestingly, physical exercise can improve cardiovascular dysfunction *via* upregulating the levels of miR-29a and miR-29c, which are associated with inflammatory cytokines released in sepsis (Soci et al., 2011).

## 4 Conclusion

Studies have shown that exercise preconditioning can improve cardiovascular, neurological, respiratory, and hepatic dysfunction in sepsis, and increase the survival of sepsis patients. Nevertheless, doubts remain about the effectiveness of this therapy in sepsis. Thus, there is a need for more clinical research to evaluate whether physical exercise can attenuate organ dysfunction in sepsis. Moreover, new knowledge is needed on the effects of post-hospital exercise on the long-term outcomes of sepsis. This knowledge can further our understanding of whether physical exercise can be a non-pharmacological treatment for sepsis.

**FIGURE 2:**
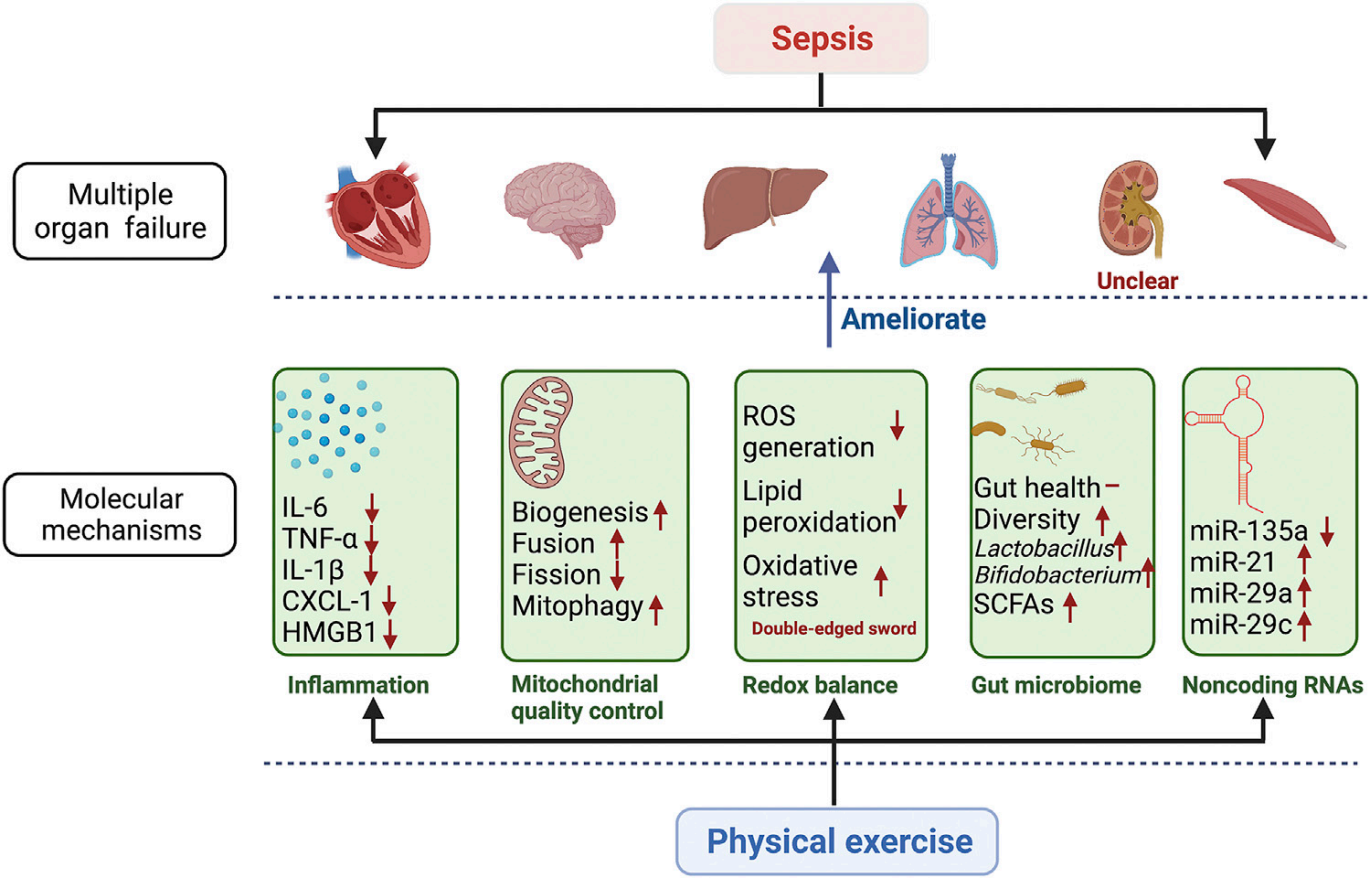
Molecular mechanisms involved in the beneficial effects of physical exercise on multiple organ failure in sepsis. The figure shows how physical exercise regulates multiple organ failure through these potential molecular mechanisms. The potential molecular mechanisms include inflammation, mitochondrial quality control, redox balance, gut microbiome, and noncoding RNAs. These potential molecular mechanisms regulated by physical exercise ameliorate sepsis-induced multiple organ failure, including respiratory, cardiovascular, neurological, hepatic, hematological, and muscle systems. The effects of physical exercise on renal dysfunction are unclear in sepsis. IL-6 interferon 6; TNF-α tumor necrosis factor α; IL-1β interferon 1β; CXCL-1 chemokine (C-X-C motif) ligand 1; HMGB1 high mobility group 1; ROS reactive oxygen species; SCFAs short-chain fatty acids; miR-135a microRNA 135a; miR-21 microRNA 21; miR-29a microRNA 29a; miR-29c microRNA 29c.

In this review, we outlined the potential mechanisms of the beneficial effects of physical exercise on sepsis (Figure 2). We illustrated that mitochondrial biogenesis, mitochondrial fusion and fission, mitophagy, systemic inflammation, redox balance, the gut microbiome, and noncoding RNA are involved. Despite existing investigations into these molecular mechanisms, many of the mechanisms associated with physical exercise and sepsis have not yet been revealed. There is a need for further research to systematically screen molecular mechanisms that are associated with physical exercise and sepsis.
